# Semaphorins as Potential Immune Therapeutic Targets for Cancer

**DOI:** 10.3389/fonc.2022.793805

**Published:** 2022-01-27

**Authors:** Jun Jiang, Fang Zhang, Yi Wan, Ke Fang, Ze-dong Yan, Xin-ling Ren, Rui Zhang

**Affiliations:** ^1^ Department of Health Service, Fourth Military Medical University, Xi’an, China; ^2^ State Key Laboratory of Cancer Biology, Department of Immunology, Fourth Military Medical University, Xi’an, China; ^3^ Department of Respiratory Medicine, Xijing Hospital, Fourth Military Medical University, Xi’an, China; ^4^ Department of Biomedical Engineering, Fourth Military Medical University, Xi’an, China; ^5^ Department of Pulmonary Medicine, Shenzhen General Hospital, Shenzhen University, Shenzhen, China

**Keywords:** semaphorins, cancer, immunotherapy, immune cell, therapeutic targets, tumor microenvironment

## Abstract

Semaphorins are a large class of secreted or membrane-bound molecules. It has been reported that semaphorins play important roles in regulating several hallmarks of cancer, including angiogenesis, metastasis, and immune evasion. Semaphorins and their receptors are widely expressed on tumor cells and immune cells. However, the biological role of semaphorins in tumor immune microenvironment is intricate. The dysregulation of semaphorins influences the recruitment and infiltration of immune cells, leading to abnormal anti-tumor effect. Although the underlying mechanisms of semaphorins on regulating tumor-infiltrating immune cell activation and functions are not fully understood, semaphorins can notably be promising immunotherapy targets for cancer.

## Introduction

Relative to traditional cancer treatments, tumor immunotherapy has shifted the paradigm for the treatment of cancer (1). Particularly, the emergence of immune checkpoint inhibitors (ICIs, such as CTLA-4 and PD-1/PDL-1 inhibitor) (2) and adoptive cell therapy (chimeric antigen receptor T cells, CAR-T) (3) represents a turning point for tumor treatment. However, due to the existence of multiple immunosuppressive mechanisms in the tumor microenvironment (TME), tumor cells can get rid of the surveillance and immune killing effects of the immune system under various immune escape pathways.

Semaphorins, initially characterized as axon guidance factors, are membrane-bound or secreted proteins that participate in cell-to-cell communication and functions (4). Semaphorins play versatile roles in pathophysiological processes, including cancer, immune diseases, and bone diseases, which can be used as novel targets for drugs for preventing or treating various diseases (5–7). There are more than 20 kinds of semaphorins in vertebrates, which can be divided into class 3–7 categories. Class 3 semaphorins are secreted proteins, whereas the others are membrane-bound proteins, and membrane-bound Class 4 semaphorins can be shed into soluble forms by proteolytic cleavage under certain circumstances (6, 8, 9).

**Figure 1:**
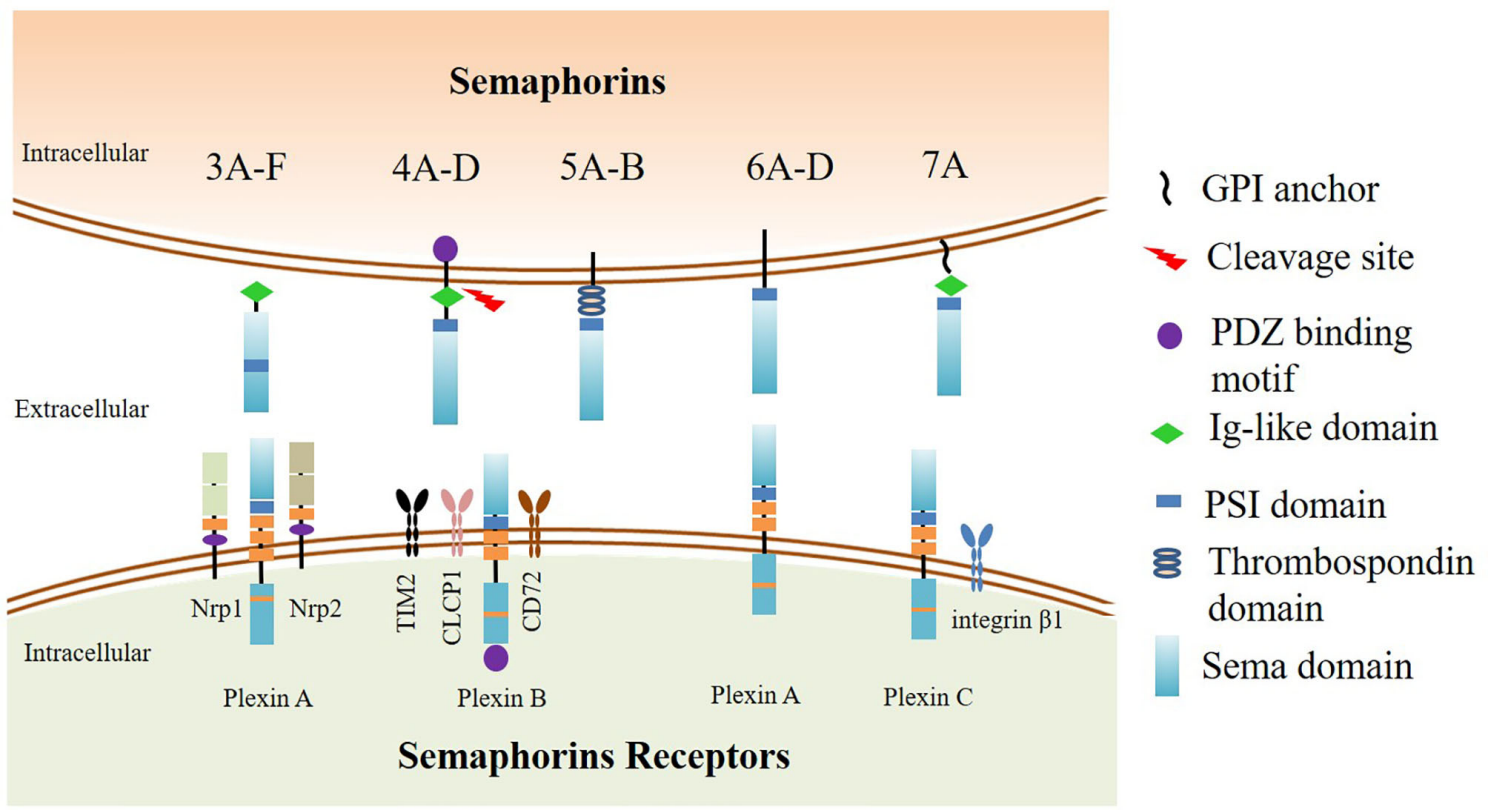
The classification and structure of semaphorins and their receptors. The upper part: Class 3 semaphorins are secreted proteins. Class 4 to 6 semaphorins are membrane-bound proteins. Sema7A is the only GPI-linked protein in the semaphorin family. The N-terminus of the semaphorins is Sema domain. Adjacent to the downstream area of the Sema domain is the plexin-semaphorin-integrin (PSI) domain. Class 3, 4, and 7 semaphorins contain an immunoglobulin-like domain located downstream to the PSI domain. Class 4 semaphorins have a PDZ binding motif. The lower part: The receptors of semaphorins. The most membrane-bound semaphorins directly bind to conservative plexins, which are classified into four classes A–D. Plexin A proteins are mainly associated with class 5 and 6 semaphorins, whereas Plexin B proteins are mainly associated with class 4 and 5 semaphorins, and Plexin C proteins are bound with Sema7A. Secreted class 3 semaphorins transmit signals requiring neuropilins (Nrps) as coreceptors. Neuropilins are divided into two subtypes, Nrp1 and Nrp2. There are a few semaphorins that require additional interactors to participate in biological activities. Sema4A binds to TIM2, Sema4B binds to CLCP1, Sema4D binds to CD72, and Sema7A binds to integrin β1.

Semaphorins contain a common “sema domain”, the domain for receptors binding. The main receptors of semaphorins are Neuropilins or Plexins families. The most membrane-bound semaphorins directly bind to conservative plexins that also contain a “sema domain”. Plexins can be classified into four classes, A–D, and transfer signals mediated by small GTPases (10), whereas soluble class 3 semaphorins transmit signals requiring neuropilins (Nrps) as co-receptors (11). Nrps are divided into two isoform subtypes Nrp1 and Nrp2. Nrp1 is essential for immune response and identified as the co-receptor of VEGF to mediate angiogenesis (12), and Nrp2 exerts a significant role in VEGF-C/D/VEGFR-3-mediated tumor lymphangiogenesis and lymphatic metastasis (13). Moreover, there are a few semaphorins that require additional receptors to participate in biological activities. For instance, Sema4A can bind to TIM2 (14), Sema4B to CLCP1 (15), Sema4D to CD72 (16), and Sema7A to integrin β1 (17) (**Figure 1**).

Accumulating evidence indicate that semaphorins are dysregulated and play versatile and multifaceted regulatory roles in several hallmarks of cancer, including angiogenesis (18), metastasis (19), tumor immune escape, and tumor-associated inflammation (20–22). Semaphorins can contribute to tumor progression by modulating immune responses between tumor cell and tumor-infiltrating immune cells in TME. The immunological function of semaphorins is widespread, mainly due to membrane-bound semaphorins or their receptors widely distributed on the surface of immune cells and tumor cells. The so-called immune semaphorins can act as attractants to regulate the recruitment of macrophages, natural killer cells (NK), dendritic cells (DCs), and cytotoxic T lymphocytes (CTL) to the TME (23). For instance, Sema3A, Sema4C, and Sema4D have been found to promote tumor progression by enrichment of tumor-associated macrophages in TME (24, 26). On the other hand, Sema3A, as a tumor suppressor, has been reported to restrict the proliferation of pro-tumoral macrophages and repress tumor growth (27). The role of Sema4A is also intricate in tumor immunity. Sema4A expression enhances B-cell infiltration, which contributes to favorable outcome for head and neck squamous cell carcinoma (28), and Sema4A expression on DCs activates CTL and exerts anti-tumor in Lewis lung cancer (29), whereas Sema4A maintains the stability and function of Tregs in melanoma (30). Due to the versatile and multifaceted regulatory roles of semaphorins in tumor-infiltrating immune cells, semaphorins with their receptors could mediate intricate cross-talking between tumor cells and the microenvironment. This review mainly illuminates the regulatory effects and potential mechanisms of representative semaphorins on tumor-infiltrating immune cells, as well as the potential application of semaphorins as therapeutic targets for tumor immunotherapy.

## Semaphorins and Tumor Associated Macrophages

Tumor-associated macrophages (TAMs, Mϕ) are main infiltrating cell groups in tumor stroma and closely associated with tumor angiogenesis, invasion, and metastasis. TAMs have two opposing phenotypes, anti-tumorigenic M1-Mϕs and pro-tumorigenic M2-Mϕs. M1-Mϕs function as inhibiting tumor progression by secreting pro-inflammatory cytokines (IFN-α/β/γ and IL-12) and chemokines (CXCL9 and CXCL10), which can attract CTL and NK cell to restrict tumor growth (31, 32). M2-Mϕs suppress tumor immunity and accelerate tumor progression by secreting immune suppressive factors, such as cytokines (TGF-β and IL-10) and chemokines (CCL2, CCL17, CCL22, and CCL24) (33, 34). TAMs can also secrete pro-angiogenic factor vascular endothelial growth factor (VEGF), placental growth factor (PLGF), and Sema4D to promote angiogenesis, and express podoplanin (PDPN, lymphatic marker) to promote lymphangiogenesis in paracrine and autocrine pathways, leading to tumor vascular and lymphatic metastasis (18, 35). Studies have shown that semaphorins play significant roles in the migration and polarization of TAMs.

### Sema3A

Sema3A, a secreted protein, plays paradoxical roles in TME in different types of tumors. In breast cancer, Sema3A is a tumor suppressor, downregulated in tumor and negatively correlated with tumor stage. *In vivo*, Sema3A overexpression increases CD11b^+^F4/80^+^ Mϕs accumulation but not CD11b^+^Ly6C^+^ monocytic cells, and reduces 4T1-3A^+^ tumor growth in immune complete BALB/c mice. Sema3A regulates intratumoral M1-Mϕs (CD11b^+^Ly6G^-^Ly6C^low^MHCII^high^) and M2-Mϕs (CD11b^+^Ly6G^-^Ly6C^low^MHCII^low^) differentiation by binding to its receptor Nrp1, and increases M1-Mϕs proliferation but represses M2-Mϕs by enhancing CSF1-mediated phosphorylation of Akt and MAPK, inducing CD8^+^ T cells and NK cells to repress tumor growth (27).

However, in Lewis lung cancer, Sema3A binding to Nrp1 and PlexinA1/PlexinA4 coreceptors promotes tumor growth by TAM infiltration and pro-tumorigenic function in hypoxic areas (24). Under the tumor hypoxia environment, Sema3A is upregulated, attracting TAMs from the vascularized and perfused area to the hypoxic area by binding to Nrp1/PlexinA1/PlexinA4/VEGFR1 (24). Interestingly, when the expression of Nrp1 on TAMs is downregulated, TAMs are stopped migrating from normoxic regions to hypoxic region by Sema3A/PlexinA1/PlexinA4-mediated stop signals. The redistribution of TAMs weakens their angiogenic and immunosuppressive ability and hinders orthotopic and spontaneous tumor growth (36, 37). In terms of glioblastoma (GBM), the expression of Sema3A is significantly higher in tumor tissues relative to adjacent normal tissues. Sema3A derived from GBM elicits TAMs (microglial cell) accumulation, and antibody blockage of Sema3A (anti-Sema3A, F11) exhibits notable tumor inhibitory effect through downregulating TAMs recruitment in patient-derived xenograft (PDX) models (38). In addition, upregulation of Sema3A boosted the phosphorylation of downstream PI3K and AKT by binding to Nrp1, and enhanced the enrichment of M2-Mϕs to promote resistance to androgen deprivation therapy in prostate cancer (39). Another study has demonstrated that blockage of Sema3A/Nrp1 could also enhance anti-tumor response by increasing M1-Mϕs and decreasing M2-Mϕs in colorectal carcinoma (40). These studies indicate that Sema3A, particularly binding to its receptor Nrp1 on TAMs, regulates the recruitment and differentiation of TAMs in TME. Targeting SEMA3A and Nrp1 has proved to be a novel approach for multiple malignances.

### Sema4C

Sema4C, a transmembrane protein, is overexpressed in multiple types of malignant tumors, including breast cancer, esophageal cancer, gastric cancer, and rectal cancer (41, 42). In breast cancer, the functions of Sema4C in macrophage recruitment contribute to tumor malignant properties. Sema4C with plexin-B2 receptor promotes macrophage infiltration in TME, and promotes tumor growth and progression by activating the NF-κB pathway to induce CSF-1 production in breast cancer (25). Additionally, Gao Qinglei found that MDA-MB-231 with shSema4C attracted few macrophages relative to empty vector control cells in *in vitro* migration assays (25). Membrane-bound Sema4C could be cleaved by matrix metalloproteinases to produce soluble Sema4C. A multicenter retrospective study demonstrated that soluble Sema4C was a potential biomarker for breast cancer diagnosis (43). Thus, not only could membrane-bound Sema4C be a promising target to macrophage for immunotherapy, but soluble Sema4C could also be a diagnostic biomarker for breast cancer.

### Sema4D (CD100)

Sema4D, also known as CD100, is a transmembrane molecule of 150 kDa of semaphorins IV subfamily, and upregulated in multiple tumor tissues, such as lung, colon, and breast cancer (44–46). Additionally, Sema4D is the first semaphorin member known to be widely expressed on immune cells (16). CD72, Plexin-B1, and Plexin-B2 are the receptors of Sema4D. CD72 is mainly expressed in immune cells and regulates immune response by combining with Sema4D, whereas Plexin-B1 and Plexin-B2 are widely expressed on endothelial cells in multiple tissues and can trigger MET tyrosine kinase signals to promote angiogenesis by interacting with Sema4D ligand (47, 48).

Zhou Yan-Bing’s research found that Sema4D and CD68 (TAMs marker) expression were significantly higher in gastric tumor tissues than that in adjacent normal tissues and correlated with histological differentiation type, TNM stage, and lymphatic metastasis by clinicopathological features analysis of 290 gastric patients (26). *In vitro*, they further found that gastric carcinoma SGC-7901 cells showed great morphological changes after non-contact co-culture of M2-Mϕs: cubic tumor epithelial cell with blunt edge and high confluence shifted to narrow interstitial cell-like shape with long spindle and less confluence. TAMs enhanced the expression of Sema4D on SGC-7901 cells, and promoted invasion and metastasis abilities of SGC-7901 cells *in vitro.* It indicated that targeting Sema4D might be able to bring favorable prognosis for gastric patients. However, anti-Sema4D treatment with a specific antibody (Mab67, Vaccinex) shrank tumor bulk and improved survival rates in pancreatic neuroendocrine cancer (RIP1-Tag2) mice in a short period, but conversely promoted lymph node metastasis consistent with an increase in TAMs after anti-Sema4D treatment (49). To further identify the mechanism of TAMs promoting metastasis, the study of Oriol Casanovas found a significant increase in stromal cell-derived factor 1 (SDF1, CXCL12, a pro-invasive molecule) after anti-Sema4D treatment through a mouse cytokine array. In the presence of anti-Sema4D antibodies, macrophages secrete SDF1, which leads to stronger tumor cell migration by binding to CXCR4 receptor.

### Sema7A

Sema7A, also known as CD108, the only GPI-linked protein in the semaphorin family, promotes neutrophil migration under hypoxia stimulation (50). Sema7A increases α1β1-integrin macrophages in viral myocarditis (51). Sema7A can recruit macrophages not only in viral infection, but also in TME. Elder and Tamburini found that Sema7A might be involved in macrophage-mediated lymphangiogenesis in breast cancer (52). Sema7A promotes macrophages podoplanin (PDPN) expression, migration, and adhesion of the lymphatic epithelial cell, resulting in breast cancer lymphatic metastasis. PDPN-expressing macrophages (PoEMs) can activate integrin β1 (Sema7A receptor) to bind to lymphatic endothelial cells expressing galectin 8 (GAL8) and cause lymphatic vessel remodeling, lymphangiogenesis (35). Lymphangiogenesis depends on PDPN-CLEC-2 (PDPN receptor) interaction and Sema7A-integrin β1 interaction. Therefore, CD68, Sema7A, and PDPN are associated with poor prognosis of breast cancer patients with lymphatic metastasis (52).

TAM infiltration, especially pro-tumorigenic M2-Mϕs, are related with poor prognosis of multiple cancer types. Sema4C, Sema4D, and Sema7A can be considered as promising biomarkers of TAM infiltration and can be used as prognostic indicators of cancer. Moreover, depletion of TAMs by Sema4D blockage to decrease M2-Mϕs recruitment and aggregation, to eliminate TAMs-associated angiogenesis and metastasis, is a potential strategy to cancer treatment. However, the strategy of TAM depletion may lead to a decline in ability of tumor antigen presentation. Therefore, reprogramming TAM polarization from M2-Mϕs to M1-Mϕs by altering Sema3A expression can be another effective approach to enhance anti-tumor effects.

## Semaphorins and T Lymphocytes

The presence of CAR-T targeting to tumor-infiltrating lymphocytes (TILs) has greatly improved clinical outcome in cancer, particularly for hematologic malignancies, but fail to effectively eliminate cancer cells. Due to insufficient expression of MHC-I or the presence of immunosuppressive signals, the anti-tumor effect of CTL is greatly compromised and displays dysfunctional states (53, 54). PD-1, CTLA-4, T-cell immunoglobulin, and mucin domain-3 protein (TIM-3), lymphocyte-activation gene 3 (LAG-3, CD223) (55), B- and T-cell lymphocyte attenuator (BTLA, CD272) (56), T-cell immunoglobulin and ITIM domain (TIGIT) (57), and V-domain Ig suppressor of T-cell activation (VISTA) (58) have been described as hallmarks of T-cell exhaustion. Semaphorins and their receptors (particularly Nrp-1) have multiple roles in T-cell responses. Nevertheless, the potential role of semaphorin/Nrp-1 in regulating immunosuppressive receptors and CTL functions is complicated.

### Sema3A and Sema3B

Recently, numerous lines of evidence indicate that the Sema3 family with Nrp-1 receptor play vital roles in inhibiting anti-tumor CD8^+^ T-cell responses (59). Sema3A and Sema3B, constitutively distributed on immune cells, binding to Nrp-1, contribute to immune escape from anti-tumor effects of CD8^+^ CTL (60).

Nrp-1 and Nrp-2 have been shown to be expressed on DCs, macrophages, and T-cell subpopulations and mainly exert pro-tumor effects (61, 62). Nrp-1, a transmembrane protein, is widely involved in cardiovascular and neuronal development, and can also regulate cancer immunology (59). Moreover, Nrp-1 is also co-receptor of VEGF and the Sema3 family (40). Nrp-1 has been characterized in different immune cellular phenotypes including macrophages, dendritic cells, and T-cell subsets, especially expressed on activated T cells and regulatory T-cell populations, but not on the resting T cells (63–65). Nrp-1^+^ Tregs are highly expressed in both TME and peripheral blood, making Nrp-1 a potential immune checkpoint target for immunotherapy (66). Those expressing high Nrp-1 CTL subset also express high PD-1^+^, with the co-expression of other T-cell inhibitory receptors like CTLA-4, Tim-3, and LAG-3 in B16F10 melanoma (59). The combination of PD-1 antibody and Nrp-1 antibody is more efficient in repressing tumor growth *in vivo*. By contrast, Nrp-2, another isoform, is comparatively less studied in T cells. The expression pattern of Nrp-2 varied in the CD4/CD8-defined subsets. Nrp-2 was upregulated in the CD4^+^CD8^+^ DP T cells and downregulated in SP CD4^−^CD8^+^ and CD4^+^CD8^−^ cells as they gradually became lineage committed (63).

Sema3A secreted from activated DCs and T cells can bind to Nrp-1 on T cells and inhibit T-cell proliferation. However, Yang Zhi-Gang has reported that Sema3A was downregulated in acute leukemia, and exogenous Sema3A could inhibit the Nrp-1 expression on Tregs and promote apoptosis in leukemia cells (67). Those studies indicated that Sema3/Nrp-1 signaling was a novel target for tumor immunotherapy (65).

### Sema4A

Sema4A, as a new class of immune regulatory molecules, is not expressed by resting T cells, but can be induced on activated T cells (14), constitutively expressed on APCs like dendritic cell and co-stimulates activation of CD4^+^ T cells. Sema4A has been found to promote Th1-cell-mediated IFN-γ production in mice, but eliciting Th2-cell-mediated IL-4, IL-5, and IL-13 production in human by binding with immunoglobulin-like transcript 4 (ILT-4) receptor (68, 69).

Regulatory T cells (Tregs) have effects on limiting immunopathology, preventing autoimmune diseases, and maintaining immune homeostasis and also negatively regulating anti-tumor immunity (70). The deletion of Tregs can induce the reduction and elimination of tumors, but may induce uncontrolled autoimmunity and even death. Sema4A interacting with Nrp1 also promotes Treg cells’ survival, stability, and function through modulation of the Akt-mTOR signaling and PTEN-Akt-FoxO axis (30). The deficiency of Nrp-1 on Tregs fails to limit autoimmunity and induces autoimmune diseases. Thus, the Nrp-1 receptor on Treg cells is dispensable for the suppression of autoimmunity and the maintenance of immune homeostasis. Sema4A–Nrp1 blockade *via* antibodies or soluble antagonists is possible to limit tumor growth by targeting Treg cells without triggering autoimmunity.

### Sema4D

CD100 has two forms, soluble CD100 (sCD100) and membrane-bound CD100 (mCD100). Both mCD100 and sCD100 have vital roles in immune response. mCD100 is constitutively expressed on the resting T cells, and can be cleaved into sCD100 by matrix metalloproteases when T cells are activated (71, 72). The function of Sema4D on CD8^+^ CTL is controversial. In HIV infection, the CTL is in lack of mCD100, leading to anti-virus capacity being disabled (73), while sCD100 enhances CTL function of virus clearance in HBV infection (74). Fan Fei-Fei found that MMP-14, sCD100 level decreased and mCD100 increased in non-small cell lung cancer (NSCLC) compared with healthy people, whereas recombinant CD100 or sCD100 upregulation by MMP-14 enhanced CTL activity by secreting IFN-γ and TNF-α. Moreover, the effect of sCD100 on CTL could be blocked by anti-CD72 antibody. Thus, it indicates that sCD100 shedding depends on the cleavage of MMP-14 and CD72 interaction and plays an important role in regulating CTL of NSCLC (75).

Evans has reported that Sema4D displays an immunomodulatory function. When Sema4D is highly expressed on the invasive margins of actively growing tumors, it influences the infiltration and distribution of leukocytes in the TME. Antibody neutralization of Sema4D disrupts this gradient of expression, enhances recruitment of activated monocytes and lymphocytes into the tumor, and shifts the balance of cells and cytokines toward a proinflammatory and antitumor milieu within the TME. This change in the tumor architecture was associated with durable tumor rejection in murine Colon26 and ERBB2(+) mammary carcinoma models (46). Recently, a Phase Ib/II study of pepinemab (anti-Sema4D) in combination with avelumab (anti-PD-L1) showed that the combination therapy was well tolerated and exerted antitumor activity in immunotherapy-resistant and PD-L1-low NSCLC patients (76). However, the function of Sema4D on Treg responses in cancer is still unknown. In ankylosing spondylitis, Sema4D inhibits Treg cell differentiation in the AhR pathway (77). Sema4D also promotes liver fibrosis in Schistosomiasis infection *via* TGF-β1 and IL-13 pathways. Sja-miR-71a in Sjaponicum egg-derived EVs can increase Treg and decrease Th1, Th2, and Th17 by directly inhibiting Sema4D (78).

Exhausted T cells not only highly express PD-1 and CTLA-4, but also highly express semaphorins and their receptors, especially Nrp-1; thus, tumor cells are compromised to immune checkpoint inhibitors and turn to self-tolerance. Moreover, the expression of semaphorins and Nrp-1 is positively correlated with PD-1 expression level. Therefore, concomitant blockade of semaphorins, Nrp-1, and PD-1 may reshape the anti-tumor function of CTL and abrogate tumor progression.

## Semaphorins and Tumor-Infiltrating B Cell

T cells are not the only immune cells capable of fighting tumor cells. Tumor-infiltrating B cells (TIL-Bs) are also important for tumor immunity. Recent studies have found that bulk of B cells are enriched in tumor tissues including lung cancer, melanoma, renal cell carcinoma, breast cancer, and head and neck squamous cell carcinoma (HNSCC) (28, 79, 80), and B cells play a dual role in the progression of cancer. On the one hand, B cells can stimulate anti-tumor immunity by antigen presentation B cell (APC-B cell) and producing IgG (Plasma cell) to mediate antibody-dependent cytotoxicity; on the other hand, regulatory B cells (B-regs) inhibit CD8^+^ T cell activity by secreting IL-10, PD-L1, and TGF-β, resulting in tumor immunosuppressive effects and tumor progression. TIL-Bs have prognostic significance and promise to be a new target to complement T-cell-based immunotherapy. The expression of Sema4D on resting B cells is low, but upregulated upon activation. Sema4D has been found to promote the survival and activation of B cells and enhance antibody production (81), but the role of semaphorins on TIL-B is rarely reported.

## Sema4A

TIL-B mainly comprise naïve B cells, germinal center (GC) B cells, plasma cells, etc. The team of Jennifer A. Wargo from the University of Texas MD Anderson Cancer Center found that B cells and tertiary lymphoid structures play an important role in tumor immunity (82). In HNSCC patients with human papillomavirus infection (HPV^+^) infection, GC TIL-Bs and tertiary lymphatic structure (TLS) are significantly increased, both of which correlate with a favorable outcome of HNSCC. Tullia C. Bruno found that the expression level of Sema4A was elevated in HPV^+^ HNSCC by scRNAseq data analysis (28). Interestingly, Sema4A upregulation was associated with GC B-cell differentiation and TLS with GC. Sema4A promote transition from naïve to GC cells, consistent with the expression of CD38 and BCL-6, a key transcription factor that regulates GC. It indicates that Sema4A may regulate the formation of GCs within TLS and B-cell maturity in TME of HNSCC patients.

The current immunotherapy mainly aims to activate CD8^+^ T cells, but the role of humoral immunity against tumor immunity is still unclear. As a component of the TME, TIL-Bs also play an important role in tumor progression (79). Sema4A is upregulated on GC TIL-Bs of HPV^+^ HNSCC and drives naïve TIL-Bs towards activated and GC phenotypes, which can be one way to complement current CD8^+^ T-cell-based immunotherapies.

## Semaphorins and Natural Killer Cells

NK cells are defined as CD3^-^CD56^+^ leukocytes and can be subdivided into functionally distinct subgroups, namely, CD56^bright^CD16^neg^ and CD56^dim^CD16^pos^ (83). The pan-specific innate immune recognition and rapid killing mechanism of natural killer cells (NK cells) make them another sharp sword in anti-tumor therapy apart from T cells. Decreased NK cell toxicity with KIR and NKG2A upregulation is associated with increased cancer incidence (84). Cytokine-induced memory-like (CIML) natural killer cells are preactivated with interleukin-12 (IL-12), IL-15, and IL-18, followed by adoptive transfer into patients with active acute myeloid leukemia (AML) and exhibit enhanced responses against leukemia target cells weeks later, in the form of IFN-γ production and cytotoxicity, indicating that CIML NK cells represent potent antitumor effector cells for leukemia immunotherapies (85, 86). However, the molecular mechanism of CIML NK cell differentiation and reactivation remains unknown.

## Sema7A

Adoptive transfer immunotherapy of NK cells in solid tumor patients is not satisfactory. One of the main challenges is the transport and infiltration of NK cells to the tumor site. Sema7A can regulate the migration of immune cells including NKs. Sema7A is widely expressed in lymphocytes and myeloid cells including CD56^bright^ NK cells. Stephanie Jost found that Sema7A is substantially upregulated on CIML NK cells after stimulation with cytokines (IL-12, IL-15, and IL-18), consistent with the expression of its ligand integrin-β1 and IFN-γ production (87). Strikingly, Sema7A blockade impairs substantial anti-tumor response mediated by CIML NK cells. These strongly indicate that Sema7A is a significant marker of NK cell maturation, and its ligand integrin-β1 contributes to CIML NK cell differentiation and activity.

NK cell-based tumor treatment strategies include strengthening activation of NK cell, blocking inhibitory signals on NK cell, and adoptive transfer of CAR-NK cell. Given that Sema7A/integrin-β1 interaction promotes CIML NK cell differentiation, Sema7A can be a potential biomarker of clinical outcomes for hematologic malignant patients involving CIML NK cell therapeutic interventions.

## Semaphorins and Dendritic Cell

Dendritic cells are a group of antigen-presenting cells (APCs). Most of the DCs in the human body are immature, expressing low levels of costimulatory factors and adhesion receptors. DCs can control the activation or suppression of T cells through costimulatory molecules CD80 and CD86 interaction with CD28 or CTLA4, respectively, in cancer (88, 89). Tumor-infiltrating DCs have often been viewed as tolerogenic or immunosuppressive (90, 91). However, the biological function of semaphorins that regulate mature and migratory phenotype of DCs is poorly defined.

### Sema3E

Sema3E has shown to modulate DC function in chlamydial infection. Relative to Sema3E wild-type mice, knockdown Sema3E expression exhibits higher bacterial burden by increasing Th2 response (IL-10), enhancing expression of PD-L1 and PD-L2 and reducing Th1/Th17 cytokine production (IL-12) (92). Another study found that Sema3E knockout exerted inhibitory effect on DC migration through regulation of CCR7 expression and augmenting PD-L2 expression, compared to Sema3E wild-type mice (93). These studies have shown that Sema3E can regulate the migration and function of DCs in inflammation. However, the role of Sema3E in DCs has not been elucidated in the TME.

### Sema4A

Sema4A is identified as a biomarker for DC activation status, especially in the human immune system (69). IL-33, as a candidate for cytokine therapies, can effectively enhance Sema4A expression and stimulate anti-tumoral cells including NK and CD8^+^ T cells (29, 94), while the mechanism of IL-33 on anti-tumor effects remains unclear. Sema4A on DC interacting with its Plexin B2 receptor on CTL can promote INF-γ production, increase the cytotoxicity of CTLs, and repress tumor growth (29). *In vivo* syngeneic mouse models, Sema4A knockdown abolishes the antitumor activity of IL-33. These results suggest that Sema4A may be an intrinsic antitumor effector of IL-33 in mice.

### Sema7A

DC migration is essential for host defense against tumor pathogens. The immature DCs have strong abilities to migrate. The study of Sonja I Buschow identified Sema7A as one of the most highly upregulated proteins upon DC maturation, adhesion, and migration in human and mouse by a large-scale proteome analysis (95). Sema7A-deficent DCs show an increased adhesion strength and lack the ability of migration in response to CCL21 by impairing the formation of actin-based protrusions. Sema7A knockdown impairs the actin cytoskeleton, resulting in enhancing the adhesion and attenuating migration ability of DCs (95).

Although Sema7A has a stimulating effect on the maturation and antigen presentation of DCs, which is beneficial for immune response, a growing number of studies have shown that Sema7A/integrin β1 is a promigratory signal and confers poor survival rate in glioma and breast cancer (96, 97). Therefore, Sema7A plays an anti-tumor effect in terms of DCs, but promotes tumor cells migration in the whole TME.

## Semaphorins and Myeloid-Derived Suppressor Cells

Myeloid-derived suppressor cells (MDSCs), distinctively expressing nitric oxide synthase (iNOS) and arginase-1 in the STAT3-dependent pathway (98), are bone marrow-derived immature heterogeneous myeloid cells in pathologic conditions such as chronic inflammation and cancer. MDSCs are progenitor cells of macrophages and DCs under normal circumstances, but exert immunosuppressive activity to T-cell function in the presence of maturation arrest (99). Semaphorin can regulate the polarization of MDSCs. Conejo-Garcia and Arindam Bhattacharyya found that semaphorins in exosomes derived from tumor mesenchymal stem cells promoted myeloid-derived suppressor cells (M-MDSCs) to differentiate to immunosuppressive M2-macrophages in breast cancer, but which kind of semaphorins was not mentioned in their research (100).

## Sema4D

MDSCs are major immunosuppressive cells in head and neck squamous cell carcinomas (HNSCCs), resulting in resistance to ICBs. However, the specific pathways of MDSC recruitment and infiltration remain to be investigated. In HNSCC, tumor cell-derived Sema4D inducing MDSC polarization corresponded with an inhibition in T-cell activation and an increase in arginase-1, TGF-β, and IL-10 production (101). Clint T. Allen found that Sema4D blockage improved responses to ICIs therapy for HNSCC patients due to repressing Ly6G^hi^Ly6C^int^ MDSCs (PMN-MDSCs) infiltration by reducing MAPK-dependent expression of chemokines (44, 102). Additionally, Sema4D mAb did not inhibit MOC1-tumor cell growth or tumor vascularity. These results indicated that anti-Sema4D antibodies enhance response of combination therapy by altering immune response not by inhibiting proliferation or angiogenesis, and highlighted that anti-Sema4D antibodies might be beneficial for patients with PD-1 inhibitor resistance.

## Conclusion and Future Perspectives

Although immunotherapy has been considered a breakthrough for hematologic cancers and solid tumors, the survival duration and life quality of patients are compromised to tumor immune evasion. Immune evasion is one of the hallmarks of cancer, which is one of the main reasons for the poor prognosis of patients. Imbalance between pro-tumor and anti-tumor immune response leads to immune escape of tumor cells. The immunosuppressive responses are generally manifested as an increase in expression in inhibitory receptors and ligands in APC cells (DCs, macrophages, and B cells), CTLs, and NK cells; an increase in tumor-infiltrating immunosuppressive cell types (M2-Mϕs, Tregs, B-regs, and MDSCs); hypoxic and acidic conditions; and an increase in pro-tumor cytokine and chemokine production. Accumulating evidence shows that semaphorins are involved in tumor evasion and progression. Semaphorins are dysregulated in multiple types of tumors, making them not only tumor prognostic predictors but also therapeutic targets. However, the function and signal pathways of semaphorins in the tumor immune environment are intricate and not yet fully elucidated.

**Figure 2:**
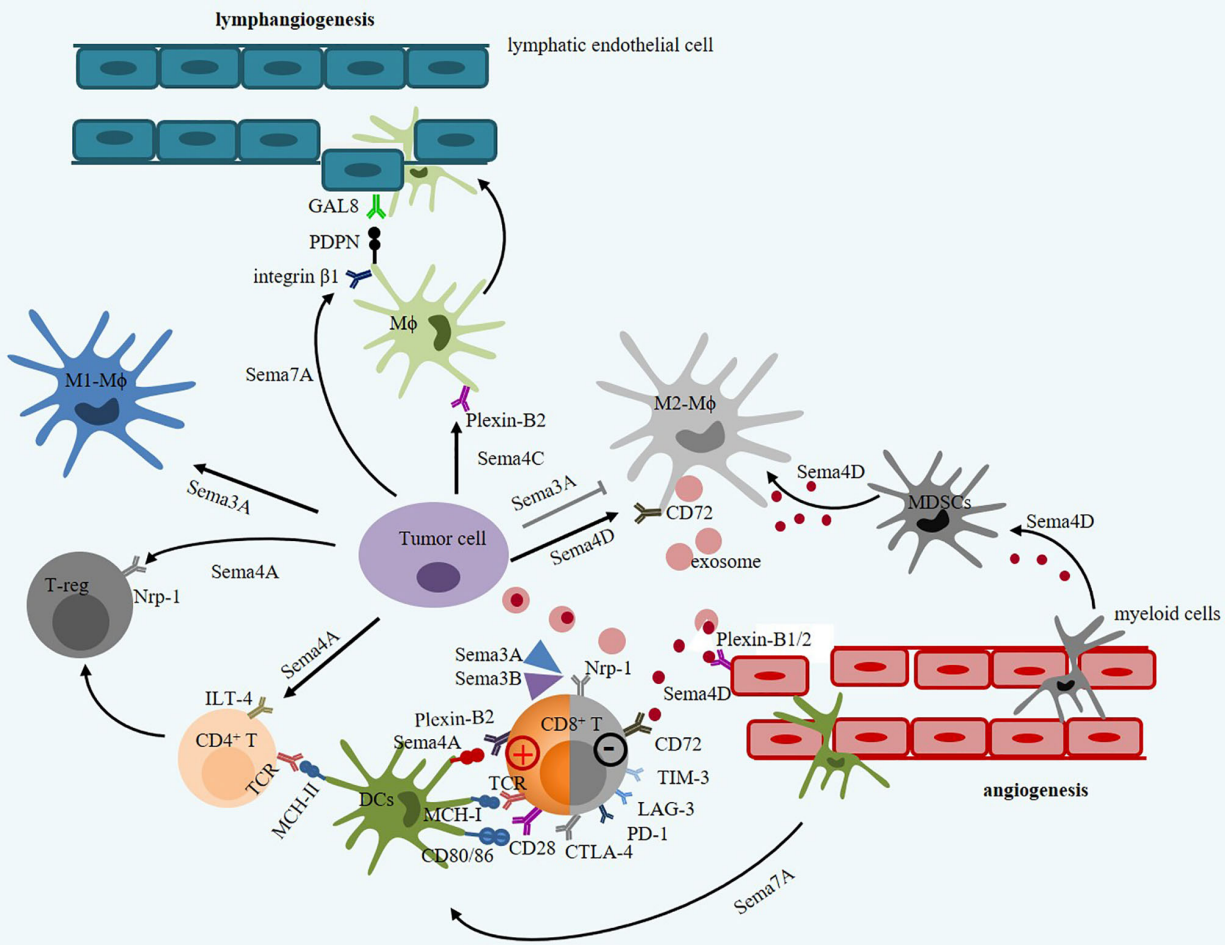
The intricate roles of immune semaphorins and their receptors in tumor microenvironment. Sema3A and Sema3B contribute to decrease in toxicity of CTL by binding to Nrp-1. However, Sema3A promotes M1-Mϕ proliferation but inhibits M2-Mϕ proliferation. Sema4A promotes Treg activation and survival *via* Nrp1 receptor, but enhance CTL vitality *via* Plexin-B2 receptor. Sema4D derived from tumor cell or M2-Mϕ promote tumor angiogenesis *via* Plexin–B1/2 receptor on endothelial cells and inhibit immune response by promoting polarization of MDSCs and inhibiting T-cell function *via* CD72. Sema4D derived from Mϕ can secrete SDF-1 (CXCL12) that mediates tumor metastasis by binding to CXCR4. Sema7A can mediate macrophages and dendritic cell migration in integrin β1 signals, and mediate tumor lymphatic metastasis through upregulating PDPN expression.

Semaphorins can act as attractants to elicit inflammation cells such as macrophages, dendritic cells, NK cells, B cells, and T cells to the TME (**Figure 2** and **Table 1**). For example, soluble Sema3A has opposite effects on the recruitment of macrophages in different types of cancer. In terms of the transmembrane Sema4 family, Sema4C and Sema4D promote macrophage recruitment and tumor progression. Sema4A promotes Treg survival and stability and accelerates tumor growth. Sema7A is constitutively distributed on resting dendritic cells, is highly upregulated on mature DCs, and is a negative regulator of T-cell responses and plays a critical role in T-cell-mediated inflammation through α1β1-integrin (103, 104). Thus, those immune semaphorins provide valuable and novel insights into immunotherapy for cancer.

**Table 1:** The roles of representative immune semaphorins in tumor microenvironment.

Semaphorins	Tumor type	Receptors	Expression	Pathway	Functions	Marker on immune cell	Ref.
Sema3A	Breast cancer	Nrp1	Sema3A downregulated	CSF1-mediated phosphorylation of Akt and MAPK	M1-Mϕs increase; M2-Mϕs decrease	M1-Mϕs: CD11b^+^Ly6G^-^Ly6C^low^MHCII^high^; M2-Mϕs: CD11b^+^Ly6G^-^Ly6C^low^MHCII^low^	(27)
	Lewis lung cancer	Nrp1 PlexinA1 PlexinA4	Nrp1 downregulated in hypoxic areas	PlexinA1/PlexinA4-dependent VEGFR1 activation	Drive TAMs toward hypoxic niches	Mϕ: F4/80^+^	(24)
	Glioblastoma	–	Sema3A upregulated	–	Elicit TAMs (microglial cell) accumulation	Microglial cell: Iba1	(38)
	Melanoma	Nrp1	Nrp1 upregulated on CD8^+^ TILs	Inhibit T-cell migration toward CXCL12 gradient	Impair CTL functions	CTL: Nrp-1^+^PD-1^hi^ CD8^+^	(59)
Sema4A	HPV^+^ HNSCC	Nrp1 PlexinD1 Tim-2	Sema4A upregulated on TIL-Bs	Correlate with BCL6 expression	Enhance germinal center TIL-Bs infiltration	TIL-Bs: CD38^+^IgD^−^ BCL6^+^Sema4A^+^	(28)
	Melanoma, colon carcinoma	Nrp1	Nrp1 expressed on Tregs	Modulate the Akt-mTOR signaling axis	Potentiate Treg-cell function and survival	Tregs: CD4^+^CD25^+^Foxp3^+^	(30)
Sema4C	Breast cancer	PlexinB2	Sema4C upregulated	Induce production of CSF-1 in plexin B2-dependent manner	Promote macrophage infiltration	Mϕ: F4/80^+^	(25)
Sema4D (CD100)	Gastric carcinoma	CD72	Sema4D upregulated	–	Promote macrophage infiltration	Mϕ: CD68	(26)
	PanNET	CD72 PlexinB2	Sema4D upregulated	Modulate the SDF1/CXCR4 signaling axis	Anti-Sema4D antibody promotes tumor migration *via* TAMs	Mϕ: F4/80^+^	(49)
	NSCLC	CD72	sCD100 decreased and mCD increased on CTLs	MMP-14 mediated CD100 shedding	Soluble Sema4D enhance CTL activity	CD8^+^ T cell subsets depend on CD45RA^+/-^, CCR7^+/-^	(75)
	HNSCC	PlexinB1	Sema4D, PlexinB1 upregulated	Reduce MAPK-dependent CXCL1 expression	Induce MDSCs polarization	G-MDSCs: Ly6G^high^Ly6C^int^ M-MDSCs: Ly6G^low^Ly6C^high^	(44)
Sema7A (CD108)	Breast cancer	Integrin β1 PlexinC	Sema7A upregulated	Drive the expression of PDPN	Promote macrophage-mediated lymphangiogenesis	Mϕ: CD68, F4/80^+^	(52)

**Figure 3:**
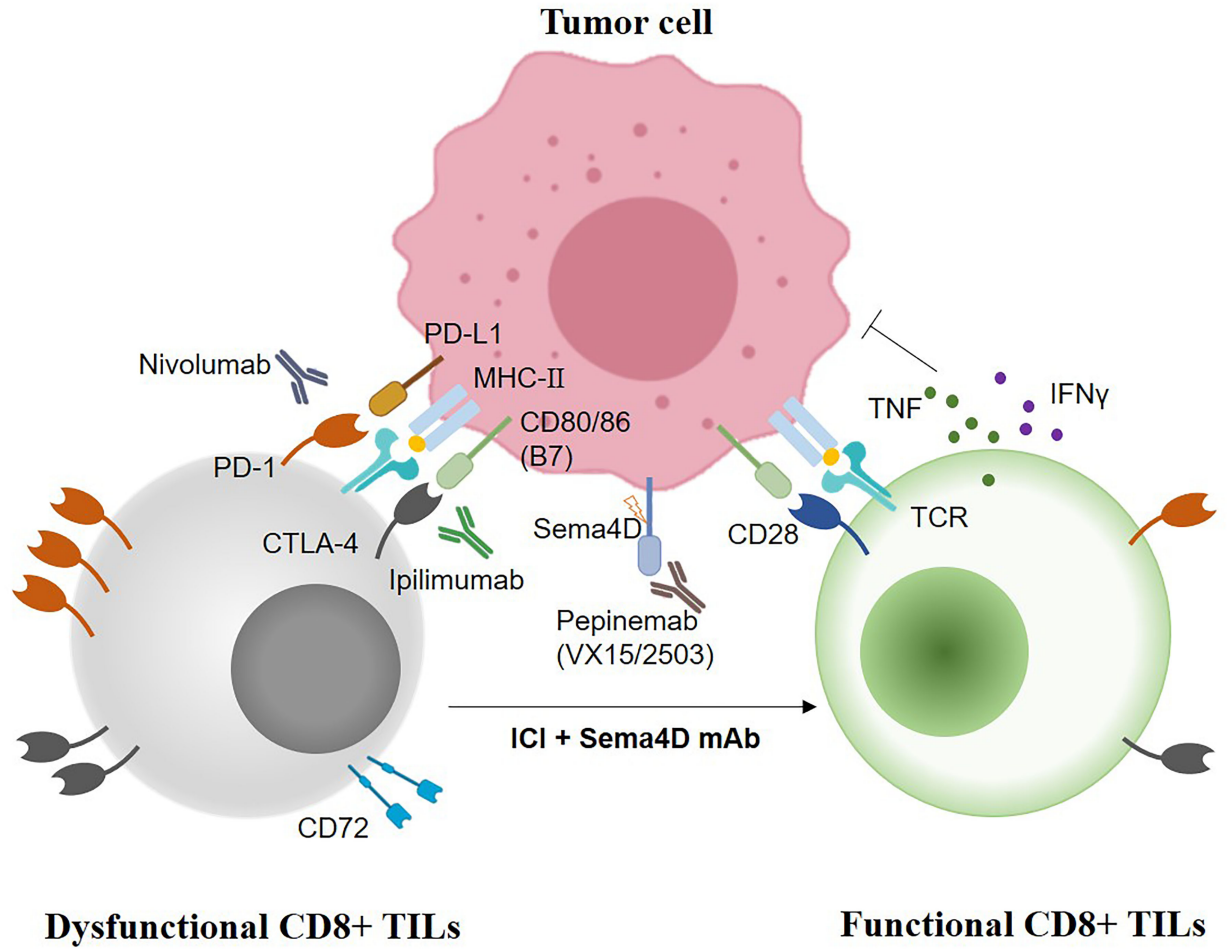
The synergistic anti-tumoral strategies of combination Sema4D mAb with immune checkpoint inhibitors (Nivolumab, PD-1 inhibitor; Ipilimumab, CTLA-4 inhibitor, Pepinemab/VX15/2503, the humanized Sema4D mAb).

Combination immunotherapy is an effective way to reshape TME and improve the therapeutic effect, particularly for immunotherapy-resistant and PD-L1 negative/low tumors. Immune semaphorin-based mAb blockade therapy has become a research hotspot. For instance, the combination of Sema4D mAb with either CTLA-4 or PD-1 inhibitor abrogates tumor growth in murine oral cancer-1 mice by inhibiting MDSC recruitment and enhances CTL infiltration (44). Recently, the combination of lgG mAb targeting Sema4D (pepinemab) with PD-L1 inhibitor avelumab has been evaluated as a safe and tolerated synthetic therapy in phase II clinical trials of immunotherapy-resistant NSCLC patients (76). Another phase I trial (NCT03425461) has been registered on ClinicalTrials.gov. to evaluate the safety and tolerability of combination of anti-SEMA4D monoclonal antibody (VX15/2503) with nivolumab or ipilimumab in patients with stage III or IV melanoma who have progressed on anti-PD1/L1-based checkpoint inhibitors (**Figure 3**). Nevertheless, the serious toxic side effects of cancer immunotherapies mainly include cytokine release syndrome (CRS) and immune effector cell-associated neurotoxicity syndrome (ICANS) (105). Advanced nanoparticle or exosome drug delivery system can transport semaphorin-based drugs to TME with specific antibody to potentially alleviate adverse effects (106). In addition to eliminating inhibitory signals in the TME, improving the immunogenicity of tumor cells to enhance CTL function is also an important strategy for immunotherapy. Adoptive transfer immunotherapy of CTL and NK cells and DC-based vaccines genetically engineered with semaphorins or their receptors of tumor cells may also be a promising cancer treatment modality. Thus, more *in vitro* studies, tumor models, and clinical trials are urgently needed to verify the effectiveness of reshaping TME and modulating immune cells by combination immunotherapy and adoptive transfer immunotherapy of immune effectors.

## Author Contributions

JJ prepared and wrote the original manuscript. FZ, YW, KF, and Z-DY edited the manuscript. RZ and X-LR revised and approved the manuscript. All authors contributed to this work and approved the submitted version.

## Funding

This work was supported by National Natural Science Foundation of China (NNSF): 81871880 and 82173046.

## Conflict of Interest

The authors declare that the research was conducted in the absence of any commercial or financial relationships that could be construed as a potential conflict of interest.

## Publisher’s Note

All claims expressed in this article are solely those of the authors and do not necessarily represent those of their affiliated organizations, or those of the publisher, the editors and the reviewers. Any product that may be evaluated in this article, or claim that may be made by its manufacturer, is not guaranteed or endorsed by the publisher.

## Abbreviations

APC, antigen presentation cell; AML, acute myeloid leukemia; BTLA, B- and T-cell lymphocyte attenuator; CTLA-4, cytotoxic T lymphocyte-associated antigen-4; CTL, cytotoxic T lymphocytes; CIML, cytokine-induced memory-like; DCs, dendritic cells; GBM, glioblastoma; GC, germinal center; HNSCC, head and neck squamous cell carcinoma; HPV, human papillomavirus; ILT-4, immunoglobulin-like transcript 4; ICANS, immune effector cell-associated neurotoxicity syndrome; LAG-3, lymphocyte-activation gene 3; MDSCs, myeloid-derived suppressor cells; Nrps, Neuropilins; NK, natural killer cells; PD-1, programmed cell death-1; PD-L1, programmed cell death-ligand 1; PLGF, placental growth factor; PDPN, podoplanin; PDX, patient-derived xenograft; PanNET, pancreatic neuroendocrine cancer; SDF1, stromal cell-derived factor 1; TME, tumor immune microenvironment; TAMs, tumor-associated macrophages; Tregs, regulatory T cells; TILs, tumor-infiltrating lymphocytes; TIM-3, mucin domain-3 protein; TLS, tertiary lymphatic structure; VEGF, vascular endothelial growth factor.
